# Poria Attenuates Idiosyncratic Liver Injury Induced by Polygoni Multiflori Radix Praeparata

**DOI:** 10.3389/fphar.2016.00386

**Published:** 2016-10-18

**Authors:** Dan Gao, Jing-Yao Pang, Cong-En Zhang, Chun-Yu Li, Can Tu, Hai-Zhu Zhang, Ming Niu, Yin Xiong, Xiao-He Xiao, Kui-Jun Zhao, Wei-Wei Gao, Jia-Bo Wang

**Affiliations:** ^1^Institute of Medicinal Plant Development, Chinese Academy of Medical SciencesBeijing, China; ^2^China Military Institute of Chinese Medicine, 302 Military HospitalBeijing, China; ^3^Pharmacy Department, Beijing Luhe Hospital Affiliated to Capital Medical UniversityBeijing, China; ^4^Department of Traditional Chinese Medicine, Beijing Friendship Hospital Affiliated to Capital Medical UniversityBeijing, China; ^5^Kunming University of Science and TechnologyKunming, China; ^6^Integrative Medicine Center, 302 Military HospitalBeijing, China

**Keywords:** Polygoni Multiflori Radix Praeparata (PM), Poria, lipopolysaccharide (LPS), idiosyncratic liver injury, inflammation, compatibility

## Abstract

The hepatotoxicity induced by Polygoni Multiflori Radix Praeparata (PM) has aroused great concern throughout the world. Hence, it is worthwhile to perform studies on the detoxification with the combined use of medicinal herbs based on the compatibility theory of traditional Chinese medicine. In this work, the rat model of PM/LPS-induced idiosyncratic liver injury was used. The effects of Poria, Licorice, and Panax notoginseng on rats of PM/LPS-induced liver injury were investigated respectively, hoping to find the most effective herbal medicine to reduce the hepatotoxicity. According to results of biochemical and histological tests, PM could induce the idiosyncratic hepatotoxicity of rats which presented modest inflammation triggered by non-injurious dose of lipopolysaccharide (LPS). We also found that the combined use of Poria and PM in the ratio of 1:2 could significantly ameliorate the PM/LPS-induced liver injury and systemic inflammation. Furthermore, UPLC/QTOF-MS-based metabolomics was performed to identify possible biomarkers and underlying biological pathways. Ten metabolites were expressed differentially among LPS, PM/LPS, and detoxification-treated groups in terms of PCA and OPLS-DA analysis, which could be potential biomarkers. MetaboAnalyst and pathway enrichment analysis revealed that alterations of these metabolites were primarily involved in three pathways: arginine and proline metabolism, primary bile acid biosynthesis and sphingolipid metabolism. This research provides systematic experimental evidences for the hepatoprotective effect of Poria against PM/LPS-induced liver injury for the first time. And these findings may help better understand the underlying mechanisms of pathophysiologic changes in PM/LPS-induced liver injury.

## Introduction

Due to the limited effectiveness and/or undesirable side effects of standard medical practice, patients often turn to alternative medicines which are often believed to be safer and sometimes better as they are “natural” and “wellness” (Seeff et al., 2001; Bent and Ko, 2004). Gradually, the increasing popularity of traditional Chinese medicine (TCM) becomes a worldwide phenomenon. Polygoni Multiflori Radix Praeparata (PM) is a well-known tonic TCM that exhibits protectiveeffects on aging, immunity, liver and kidney damage, hyperlipidaemia, and cancer (Lee et al., 2012; Lin et al., 2015; Huo et al., 2016). However, several cases of PM-induced hepatotoxicity have been reported, ranging from moderate elevations of liver enzymes levels, liver failure requiring liver transplantation to even death (Jung et al., 2011; Wang et al., 2015). Recently, we found that PM could lead to the failure of liver microcirculation perfusion (Chang et al., 2014), while it is still difficult to establish the direct causal relationship between the hepatic injury and complex constituents even in one single herbal medicine. It might be more practical to find remedies to reduce the toxicity rather than delving too deep into possible causes of hepatic injury.

The compatibility of TCM is the essence of Chinese formulae, which refers to the combination of two or more herbs based on their properties and clinical assessments. The combined herbs will have a better therapeutic effect and fewer side effects than individual herb or chemical compound (Wang et al., 2012). Poria is the sclerotium of *Poria cocos* (Schw.) Wolf. It has long been used as a sedative and diuretic recorded in An Outline Treatise of Medical Herbs, “Compendium of Materia Medica”, as “Poria is the key foundation of PM”, which can guide and support the actions of the formula to target the pathogenetic organs or improve therapeutic efficacy (Rios, 2011). Licorice, another traditional TCM herb, was claimed to coordinate the drug actions in a prescription as well as minimize the toxicity of PM when combined (Chang et al., 2015). Panax notoginseng is also recommended as a typical medicine to invigorate blood (Li H.Q. et al., 2015; Hung and Wu, 2016). However, there were few reports on the herb improving liver failure. Although the combined used of PM and those herbs was recorded in ancients books, indicating the great potential of detoxification of the selected herbs based on the compatibility theory, modern researches with stringent and scientific evidences for such combination are insufficient.

Our recent study indicated that modest inflammatory episode induced by non-injurious bacterial lipopolysaccharide (LPS) potentiated PM-induced liver injury of rats, suggesting that inflammation could be a factor for idiosyncratic drug toxicity (Li C.Y. et al., 2015; Tu et al., 2015). Thus, LPS model was used to evaluate the idiosyncratic hepatotoxicity induced by PM and detoxification by selected herbs on the basis of TCM compatibility in this study.

With the development of “omics” sciences, metabolomics has been developed to be a powerful approach in biomarker discovery and disease diagnosis (Wishart, 2016). It reveals whole metabolic profile changes of living systems in response to disease, pharmacological treatment or toxicological insult based on the comprehensive analysis of low molecular weight metabolites in biological systems (Zhang et al., 2015; Guma et al., 2016). Metabolomics is also compatible with the integrated and systemic feature of TCM, i.e., being composed of multi-components that work as a holistic system in the treatment of disease. Indeed, metabolomics is increasingly applied to understand the toxicity and mechanisms responsible for the efficacy of TCM (Liu et al., 2013; Zhang et al., 2015). Here, LPS-stimulated rats were applied as the model to study PM-induced idiosyncratic liver injury. On this basis, the combined uses of PM with Poria, Licorice or Panax notoginseng were investigated, respectively, to determine the optimal combination for reducing hepatotoxicity. Furthermore, UPLC/QTOF-MS-based metabolomics was performed to identify possible biomarkers and biological pathways that might be involved in. We hope to screen herbs reducing the liver injury caused by PM/LPS and overall understand the underlying molecular mechanisms of PM with the screened herb against liver injury of rats, providing further support for the treatment of hepatic injury attributing to PM. This study is the first systematic analysis of TCM compatibility to reduce liver injury induced by PM/LPS with endophenotype and metabolism researches.

## Materials and Methods

### Chemicals and Reagents

Lipopolysaccharide derived from Escherichia coli serotype 055:B5 was obtained from Sigma–Aldrich (Lot 113M4068V; St. Louis, MO, USA). Sodium pentobarbital was purchased from the Sigma–Aldrich (St. Louis, MO, USA). Alanine transaminase (ALT), aspartate aminotransferase (AST), and total bile acid (TBA) kits were purchased from the Jiancheng bioengineering institute (Nanjing, China). Enzyme-linked immunosorbent assay (ELISA) kits for rat interleukin 1 beta (IL-1β), interleukin 6 (IL-6), interleukin 12 beta (IL-12β), interleukin 18 (IL-18), interleukin 27 (IL-27), interferon gamma (IFN- γ), and tumor necrosis factor-α (TNF-α) were obtained from Cloud-Clone Corp. (Houston, TX, USA). Proteinase K was purchased from Sigma Chemical Company (Buchs, Switzerland). HPLC-grade methanol and acetonitrile were purchased from Merck (Darmstadt, Germany). Deionized water was prepared using a Milli-Q water purification system Millipore (Bedford, MA, USA). Other chemicals were of analytical grade and their purity was above 99.5%.

### Animals

Male Sprague-Dawley (S.D.) rats, aged 6-8 weeks and weighing 180 ± 20 g, were purchased from the Laboratory Animal Center of the Academy of Military Medical Sciences (License No. SCXK 2007-004). Animals received food and water ad libitum under standard husbandry conditions (22 ± 2°C temperature, 60-80% relative humidity and 12 h photoperiod). After one week of acclimatization, rats were grouped for experiments. All animal experiments were conducted in the Laboratory Animal center of the 302 Military Hospital. Rats received humane care in accordance with the National Institutes of Health guide for the Care and Use of Laboratory Animal, and procedures were approved by the Committee on the Ethics of Animal Experiments of the 302 Military Hospital.

### Preparation of Herb Extracts

Decoction pieces of PM were extracted twice with 8 volumes of 50% (V/V) ethanol by cold soak for 24 h. The combined extract was filtrated (15-20 μm) and concentrated under the negative pressure, freeze-dried to yield a brown ethanol extract with extraction rate of 15.7%. Decoction pieces of Poria were extracted twice with 12 volumes of boiling water under refluxing for 100 min. The collected extract was evaporated by water-bath, and then lyophilized. The extraction rate of Poria was 2.28%. Decoction pieces of Licorice were extracted with 10 volumes of boiling water using reflux method for two times with extraction length of 1 and 0.5 h, respectively. The combined extract was filtered and concentrated by reducing pressure, and dried by lyophilization. The extraction rate of Licorice was 23.58%. Decoction pieces of Panax notoginseng were extracted with 10 volumes of boiling water using reflux method for two times with extraction length of 2 and 1 h, respectively. Combined filtrates were condensed by decompressing and freeze-dried. The extraction rate of Licorice was 37.17%.

### Experimental Design

Herbal compatibility has long been demonstrated to be a reliable choice (Wang et al., 2012; Lam et al., 2015). Some promising candidates including Poria, Licorice, and Panax notoginseng have been selected to reduce the PM-induced liver injury. According to previous studies, PM combined with middle dose of Poria has been found to alleviate liver injury effectively. Furthermore, in aiming to gain a better insight into detoxification metabolism by herb compatibility and identify possible biomarkers for predicting PM-induced liver injury, the comprehensive and untargeted plasma metabolomics approach along with pattern recognition analyses was designed to characterize potential targets, determine the possible pathways and infer the biological processes by UPLC-Q-TOF-MS.

#### Detoxification by Compatibility Test

Rats were randomly divided into thirteen groups (*n* = 9 per group): control group (Ctr), LPS model group (LPS), PM-treated group (PM), Poria-treated group (Por), Licorice -treated group (Lic), Panax notoginseng-treated group (Pan), LPS group treated with PM (LPM), LPS group treated with Poria (LPor), LPS group treated with Licorice (LLic), LPS group treated with Panax notoginseng (LPan), LPS group co-treated with PM + Poria (LPP), LPS group co-treated with PM + Licorice (LPL), LPS group co-treated with PM + Panax notoginseng (LPN), coming down to three types, namely: control, model and herb groups. After 12 h of fasting with free access to water, rats were received intragastric administration of water (as control) or 1.08 g/kg PM, 0.81 g/kg Poria, 0.81 g/kg Licorice, 0.81 g/kg Panax notoginseng dissolved in water (as herb groups according to the treatment commonly used in clinic). Three hours later, rats were given 2.8 mg/kg LPS solution to model groups (with LPS groups) by tail intravenous injection. Dose of LPS was selected according to previous dose-response studies that did not cause a significant increase of ALT and AST activity (Li C.Y. et al., 2015). Seven hours later, rats were anesthetized with sodium pentobarbital (50 mg/kg i.p.) to collect samples.

#### Compatible Proportion Test

Rats were divide into the following nine groups (*n* = 9) randomly: control group (Ctr), LPS model group (LPS), LPS group treated with PM (LPM), LPS group treated with high dose of Poria (LHP), LPS group treated with middle dose of Poria (LMP), LPS group treated with low dose of Poria (LLP), LPS group co-treated with PM + high dose of Poria (LPHP), LPS group co-treated with PM + middle dose of Poria (LPMP), LPS group co-treated with PM + low dose of Poria (LPLP). Rats fasted for 12 h only with water ad libitum were received intragastric administration of water only as control and 1.08 g/kg PM, high dose of Poria (1.08 g/kg), middle dose of Poria (0.54 g/kg), low dose of Poria (0.27 g/kg) for the compatible proportion of PM and Poria 1:1, 2:1, and 4:1. Experiment was processed on the same way as before.

### Sample Collection

Rat plasma was collected with a syringe containing sodium citrate by drawing blood from the vena cava under anesthesia, then stored at -80°C for further analysis. The liver tissues were either stored with RNA store reagent overnight at the 4°C, then preserved at -80°C for PCR or excised and immersed immediately in 4% paraformaldehyde to fix for 4-6 h at room temperature followed by overnight immersion in 15% sucrose.

### Plasma Biochemical Determination

Hepatic parenchymal cell injury was estimated by evaluating plasma ALT, AST activity, and TBA content using the Hitachi clinical analyzer 7020 (Hitachi High-Technologies Co., Japan). Inflammatory microenvironment was assessed by measuring plasma inflammatory cytokines including IL-1β, IL-6, IL-12β, IL-27, IFN- γ, and TNF-α content using ELISA kits according to the manufacturer’s protocol.

### H&E Staining and TUNEL Assay

The liver samples were embedded in paraffin after fixation, and tissue sections (5 μm) were cut and processed for histological and immunohistochemical analyses. H&E staining was performed according to the standard H&E protocol.

Terminal deoxynucleotidyl transferase dUTP nick end labeling (TUNEL) assay identified apoptotic cells by detecting DNA fragmentation through a combination of enzymology and immunohistochemistry techniques. It was performed using the *In Situ* Cell Death Detection Kit, POD (Roche, Indianapolis, IN, USA) based on the instructions of the manufacturer. Detailed operations can be acquired in the Supplementary information. Cells stained by TUNEL were captured with fluorescence microscopy (Nikon Eclipse Ti-SR) equipped with a digital camera (Nikon DS-U3).

### RNA Isolation and Real-Time RT-PCR

Liver tissue was disrupted in liquid nitrogen, and total RNA was prepared using an RNeasy Mini Kit (Qiagen, Hilden, Germany) which included DNaseI digestion to remove any contaminating genomic DNA according to the manufacturer’s instruction. The purity and integrity of isolated RNA were determined by measuring the relative absorbance at 260 and 280 nm, respectively. Only RNA samples with 260/280 ratio between 1.8 and 2.1 were used for subsequent analyses.

For Reverse Transcriptase-real time quantitative Polymerase Chain Reaction (RT-qPCR), 1 μg of total liver RNA was reverse-transcribed to cDNA using RevertAid^TM^ First Strand cDNA Synthesis Kit (Thermo Scientific, USA) in 20 μl following the manufacturer’s protocol. After 1:25 dilution, 5 μl of the cDNAs were used as templates in Maxima SYBR Green qPCR (Thermo Scientific, USA) assays with a ABI 7500 Real-Time PCR System and 7500 System Software (Applied Biosystems, Alameda, CA, USA) to analyze IL-1β, IL-6, IL-12, IL-27, IFN-γ, and TNF-α gene expression, with conventional AB cycling parameters (40 cycles of 95°C,15 s, 60°C, 1 min). The melting curves were analyzed at 60-95°C after 40 cycles to determine to purity. Glycer-aldehyde 3-phosphate dehydrogenase (GAPDH) was used as an internal reference and each RT-qPCR analysis was performed in triplicate. Primer sequences were designed using Primer Express shown in **Supplementary Table S1**.

### Plasma Preparation and UPLC-MS Analysis

Prior to the analysis, 300 μl of plasma samples (Ctr, LPS, LPM, LPMP groups) were put into individual 2-ml microcentrifuge tubes after being thawed at room temperature. All samples were extracted by adding 900 μl of acetonitrile, vortex-mixed, centrifuged subsequently for 10 min at 21,000 *g* at 4°C. Each supernatant was carefully separate into vials and filtered by 0.22 μm microfiltration membrane for metabolomics analysis. Metabolomics was performed on Agilent Technology iFunnel 6550 Q-TOF LC/MS. Chromatographic separation and mass spectrometry were described in the Supplementary information.

### Identification of the Metabolites and Metabolic Pathway Analysis

Endogenous metabolites that contributed to the classification were found by variable importance in the projection (VIP) values, which showed the importance of each variable to the classification. Only VIP values >1 were selected and used for further data analysis. With regard to the identification of biomarkers that were selected by changing significantly (*p* value <0.05 and folder change >2), the ion spectrum was matched with the structure message of metabolites acquired from available biochemical databases, such as METLIN^1^, HMDB^2^, and KEGG^3^. The pathway analysis of potential biomarkers was performed to determine the relevant metabolic pathways with MetaboAnalyst 3.0^4^ based on the library of *Rattus norvegicus* (rat).

### Statistical Analysis

All the data were expressed as mean ± standard errors of the mean (SEM) and analyzed using the Statistical Package for the Social Sciences of Windows, version 17.0 (SPSS Inc, Chicago, IL, USA). An ANOVA with Duncan’s multiple-range test (*p* < 0.05) was used for statistical analysis.

Metabolite profiles from the UPLC-MS analysis were converted into Agilent MassHunter Workstation LC-TOF and QTOF Acquisition software (B.02.01) for data acquisition. All chromatographic data from plasma was processed by the freely available software package MZmine 2.20 (http://mzmine.sourceforge.net/), which performed peak noise removal, peak detection, and alignment in an automated and unbiased way using mzdata file from Agilent Mass Hunter Workstation Data Acquisition. The intensity of each ion was normalized with respect to the total ion count to generate a data matrix, and all these data matrices were introduced to the SIMCA-P 11.0 version (Umetrics AB, Umea, Sweden) for multivariate statistical analyses including principal component analysis (PCA) and orthogonal partial least-square-discriminant analysis (OPLS-DA) which was utilized to validate the PCA model and identify the differential metabolites. Pathway analysis was performed by the combination of a free web-based tool MetaboAnalyst and the topology with a powerful pathway enrichment analysis.

## Results

### Poria Attenuates Liver Injury Induced by PM-treated LPS Groups

**FIGURE 1:**
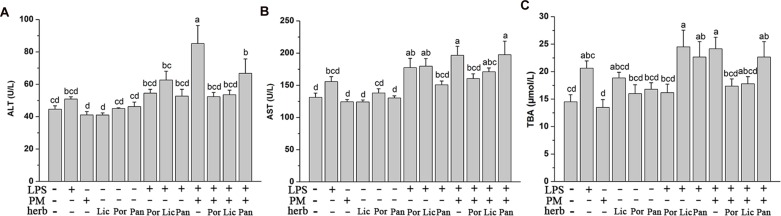
**The Plasma biochemical indicators in the absence and presence of LPS with PM and herbs including Poria (Por), Licorice (Lic), Panax notoginseng (Pan).** Rats were firstly treated with various herbs. After three hours, groups with LPS received 2.8 mg/kg LPS at caudal vein. **(A,B)** Blood samples were taken at 7 h after the administration of LPS, and plasma ALT and AST levels were measured. **(C)** Plasma TBA content were measured in the same conditions. The results were expressed as mean ± SEM (*n* = 9). Mean values followed by the same letter are not significantly different (*P* < 0.05) as determined by Duncan’s multiple-range test.

Firstly, to compare the detoxification effects of different herbs, we measured the plasma ALT, AST activity, and TBA content reflecting functional liver damage. As **Figure 1** indicated, the ALT, AST activity, and TBA content did not increase markedly in the LPS group compared to the Ctr, which proved that the dose of LPS did not cause the significant liver injury in rats at the time of sacrifice. In the meantime, rats consuming these four herbs alone almost did not alter the liver function which was indicated by the similar values of plasma ALT, AST, and TBA compared to the Ctr. However, the average concentration of plasma ALT, AST, and TBA were generally increasing after injection of LPS. This elevation was further exacerbated in LPM groups compared to those in the Ctr or LPS alone (*P* < 0.05), indicating the severity of hepatic injury and cholestasis caused by PM/LPS. Afterwards, we further focused on studying the detoxification effect of PM + Poria co-treated with LPS (LPP), PM + Licorice with LPS (LPL), and PM + Panax notoginseng with LPS (LPN), respectively. As shown in **Figure 1**, plasma AST and TBA in LPN decreased slightly compared to the LPM group. However, when compared to the Ctr, LPN group showed significant increase in plasma ALT, AST, and TBA. As for the compatibility of Licorice, levels in LPL group were lower in three biochemical parameters than the LPM group. There were even no significant differences of ALT and TBA compared to the Ctr. Surprisingly, LPP group showed markedly lower in plasma ALT, AST, and TBA than LPM group, and no significant difference was observed compared to the Ctr. Together, these data demonstrated Poria was more efficient than either Licorice or Panax notoginseng in relieving liver injury induced by PM based on LPS.

### Compatible Proportion of PM and Poria as 2:1 Suppresses Liver Injury the Best

**FIGURE 2:**
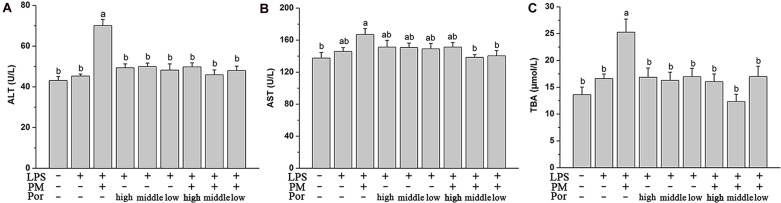
**The Plasma biochemical indicators in different dose of Poria compatible test, specifically shown as control group (Ctr), LPS model group (LPS), PM treated with LPS group, high dose of Poria treated with LPS group, middle dose of Poria treated with LPS group, low dose of Poria treated with LPS group, PM + high dose of Poria co-treated with LPS group, PM + middle dose of Poria co-treated with LPS group, PM + low dose of Poria co-treated with LPS group.** Rats were firstly treated with different compatible proportion of herbs before three hours received LPS 2.8 mg/kg at caudal vein. **(A,B)** Blood samples were taken at 7 h after the administration of LPS, and plasma ALT and AST levels were measured. **(C)** Plasma TBA content were measured in the same conditions. The results were expressed as mean ± SEM (*n* = 9). Mean values followed by the same letter are not significantly different (*P* < 0.05) as determined by Duncan’s multiple-range test.

As expected, plasma aminotransferase ALT and AST levels and TBA content increased significantly after LPS administration in LPM group. In rats co-treated PM with different proportions of Poria, the elevation of these biochemical markers were all significantly attenuated to different extends, especially with the dose of Poria 0.54 g/kg, which could fully rescue the levels of biochemical markers to normal (**Figure 2**). We concluded that this moderate proportion (2:1) of PM-to-Poria worked best in relieving liver injury induced by PM based on LPS.

### Both Compatible Proportion of PM and Poria as 2:1 and 4:1 Prevent the Elevation of PM/LPS-Induced Systemic Inflammation

**FIGURE 3:**
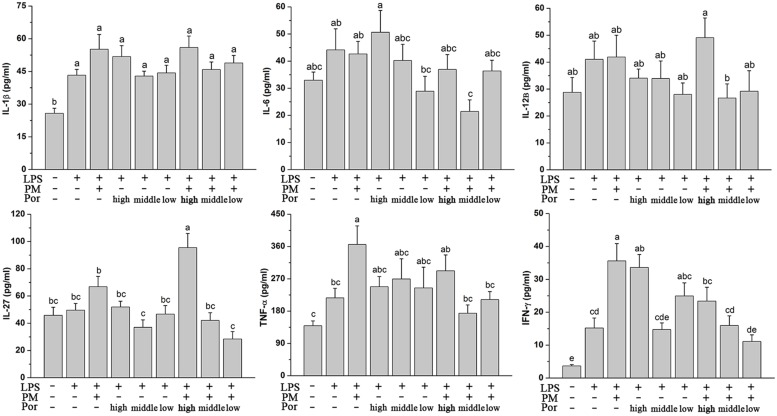
**Plasma inflammatory cytokine levels determined by ELISA.** Groups and methods of administration were the same as described in **Figure 2**. The data were expressed as mean ± SEM of nine rats. Mean values followed by the same letter are not significantly different (*P* < 0.05) as determined by Duncan’s multiple-range test.

Polygoni Multiflori Radix Praeparata led to liver injury in the presence of acute LPS and LPS played a fundamental role in systemic inflammatory response and multiple organ dysfunctions including liver. We thus hypothesized that PM/LPS might enhanced the production of inflammatory cytokines whereas Poria could counteract these effects. Changes in plasma cytokines collected from PM/LPS groups with different proportions of Poria were shown in **Figure 3**. LPS challenge caused increases in levels of the inflammatory cytokines including IL-1β, IL-6, IL-12β, IL-27, TNF-α, and IFN-γ compared with the Ctr, and among which the changes of IL-1β and IFN-γ showed significant differences (*P* < 0.05). Compared with the LPS-alone group, TNF-α and IFN-γ increased markedly while IL-1β and IL-27 increased slightly in the LPM group. Different doses of Poria-treated with LPS almost did not result in any significant changes in inflammatory cytokines levels compared to LPS group. By contrast, middle dose or low dose of Poria and PM co-treated with LPS ameliorated systemic inflammation, which was well demonstrated by significantly decreased levels of TNF-α and IFN-γ.

### Poria Reduced PM/LPS-Induced Intrahepatic Inflammatory Cytokines mRNA Expression

**FIGURE 4:**
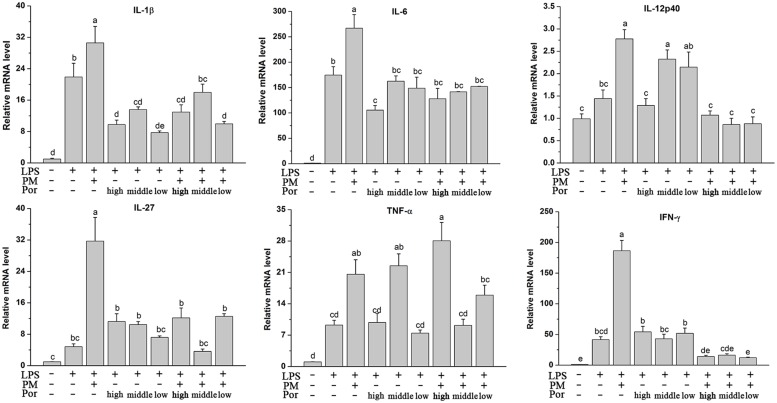
**Plasma inflammatory cytokine levels determined by RT-qPCR.** Groups and methods of administration were the same as described in **Figure 2**. The data were expressed as mean ± SEM of nine rats. Mean values followed by the same letter are not significantly different (*P* < 0.05) as determined by Duncan’s multiple-range test.

Plasma cytokine levels are not representative enough for the assessment of inflammatory processes within the liver, possibly due to the stronger influence directly from the blood stream such as leukocytes to the plasma cytokine levels (Ohira et al., 2009). Therefore, we determined the capacity of Poria to prevent the intrahepatic expression of inflammatory cytokines based on the PM/LPS model by RT-qPCR. Interestingly, although Poria did not distinctly change the PM/LPS-induced systemic inflammation release of different cytokines detected by ELISA, i.e., IL-1β, IL-6, IL-12β, and IL-27, Poria significantly inhibited intrahepatic expression of all those inflammatory cytokines including IL-1β, IL-6, IL-12p40, IL-27, TNF-α, and IFN-γ. As **Figure 4** indicated, cytokines mRNA levels increased markedly (*P* < 0.05) in LPM groups compared to LPS group. When Poria and PM were co-treated with LPS, most of cytokines mRNA expressions were significantly lowered except for the TNF-α in high dose of Poria (1.08 g/kg) to the PM/LPS-induced increase (LPHP). The levels of IL-12p40, IL-27, TNF-α, and IFN-γ were reversed to normal level by the treatment of PM and Poria 2:1which did somewhat better than that of 4:1.

### Hepatic Histopathology and Apoptosis

**FIGURE 5:**
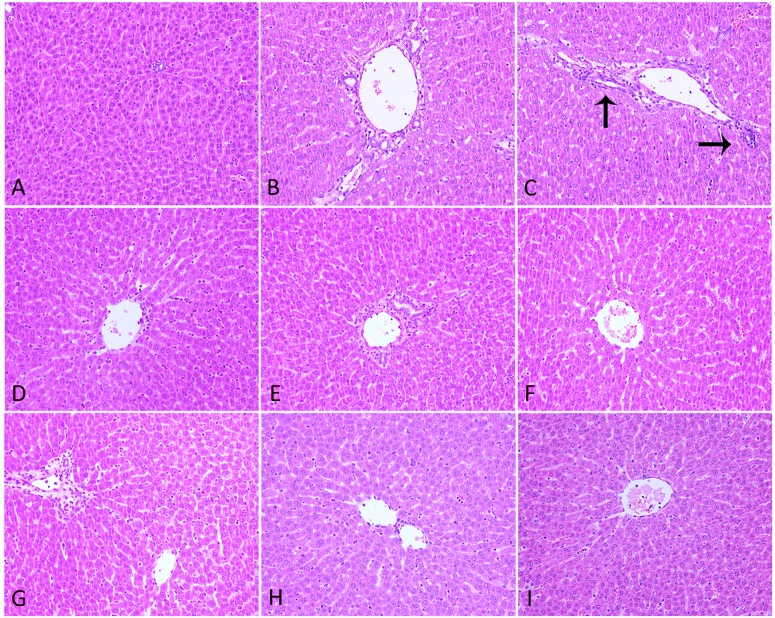
**Histopathological damage in rat liver.** **(A)** Control group (Ctr), **(B)** LPS model group (LPS), **(C)** PM treated with LPS group (LPM), **(D)** high dose of Poria treated with LPS group (LHP), **(E)** middle dose of Poria treated with LPS group (LMP), **(F)** low dose of Poria treated group with LPS (LLP), **(G)** PM + high dose of Poria co-treated with LPS group (LPHP), **(H)** PM + middle dose of Poria co-treated with LPS group (LPMP), **(I)** low dose of Poria co-treated with LPS group (LPLP). Liver sections were collected at 7 h after LPS tail intravenously (HE stained, ×200 magnification).

H&E staining analysis of liver sections revealed intact lobular architecture and normal hepatocyte structure in the Ctr (**Figure 5A**). Rats from LPS-treated alone group showed evidence of mild pathological alteration such as moderate invasion of inflammatory cells in portal area around the vessels, low level of proliferation and enlargement of Kupffer cells, indicating the activation of inflammatory signaling pathways (**Figure 5B**). In Poria/LPS-treated animals, hepatic sinus expansion, mild inflammatory cell infiltration and perivascular cells in irregularly vacuolar degeneration were observed (**Figures 5D–F**). However, there were significant pathological changes in PM/LPS groups in following manners: (a) hepatic sinus expanded significantly near the central vein; (b) part of the hepatocytes showed visible swelling; (c) some cells have no nuclei even dot necrosis; and d) high level of inflammatory cells infiltration in portal area around the vessels (**Figure 5C**). Interestingly, the combination of middle- and low-dose of Poria and PM prior to LPS exposure ameliorated these pathological changes, resulted in significant morphological protection against PM/LPS induced liver damage. However, the administration of high dose of Poria did not significantly change the histological appearances of livers compared with the PM/LPS group (**Figures 5G–I**).

**FIGURE 6:**
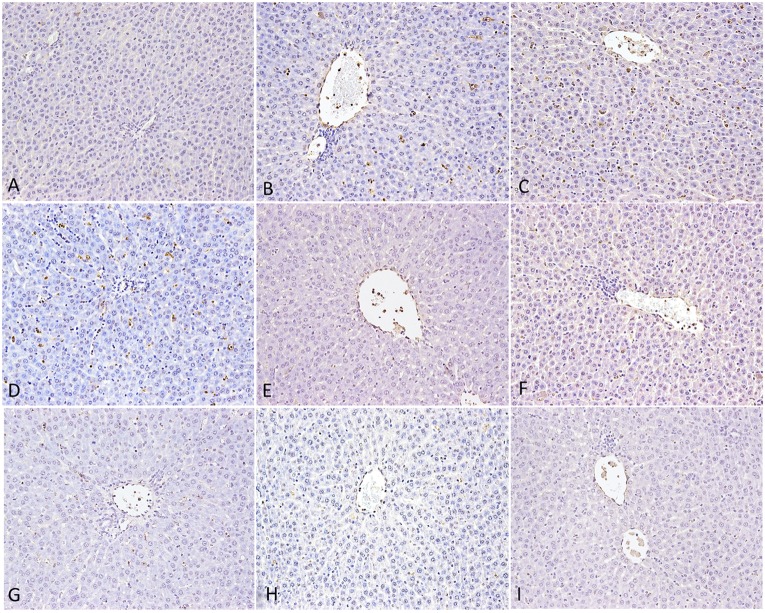
**Hepatocyte apoptosis in rat by TUNEL assay.** **(A)** Control group (Ctr), **(B)** LPS model group (LPS), **(C)** PM treated with LPS group (LPM), **(D)** High dose of Poria treated with LPS group (LHP), **(E)** Middle dose of Poria treated with LPS group (LMP), **(F)** Low dose of Poria treated group with LPS (LLP), **(G)** PM + high dose of Poria co-treated with LPS group (LPHP), **(H)** PM + middle dose of Poria co-treated with LPS group (LPMP), **(I)** low dose of Poria co-treated with LPS group (LPLP). Liver sections were collected at 7 h after LPS tail intravenously.

To confirm that Poria prevented liver injury, we performed TUNEL assays on liver sections (**Figure 6**). We did not observe any TUNEL-positive hepatocytes in the Ctr, whereas following LPS treatment, rat liver displayed some. Furthermore, a large number of cells showed TUNEL-positive in PM/LPSwith the clearly vacuolar degeneration. LPS group treated with high-dose of Poria led to more apparently positive TUNEL reaction than middle- and low-doses, suggesting that high-dose of Poria might aggravate hepatocyte apoptosis in the presence of LPS. When we administered three different doses of Poria with PM before LPS, both middle- (0.54 g/kg) and low-doses (0.27 g/kg) of Poria decreased hepatocyte apoptosis dramatically, and the middle-dose performed better. Numbers of apoptotic hepatocytes were similar between LPHP group and LPS. Taken together, these results indicated that the compatible proportion of PM and Poria 2:1 attenuated PM/LPS-induced hepatocyte apoptosis more effectively than other two doses.

### Metabolic Profiling and Biomarkers Identification

Metabolic profile of plasma samples was acquired using UPLC-QTOF/MS in positive and negative ESI modes and low molecular weight metabolites were represented as chromatographic peaks. The data containing the retention time, peak intensity and exact mass were imported into the Masslynx^TM^ software for multiple statistical analyses. Both PCA and OPLS-DA could be taken due to their ability of coping with highly multivariate, noisy, collinear and possibly incomplete data.

**FIGURE 7:**
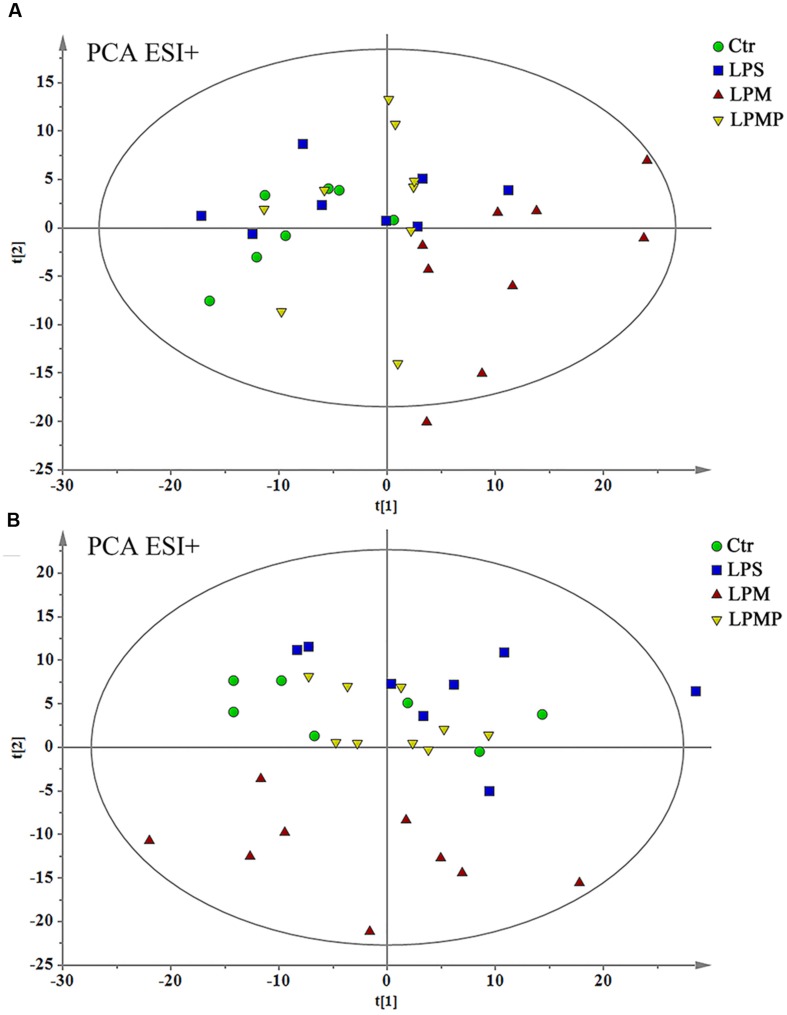
**Principal component analysis scores plot of comparing Ctr, LPS, LPM, and LPMP groups in positive ESI modes **(A)** and negative ESI modes **(B)****.

As the compatible proportion of PM and Poria 2:1 suppressed liver injury induced by PM/LPS worked the best, the Ctr, LPS, LPM, and LPMP groups were specifically selected to get a classification. The PCA analyses were performed as an unsupervised statistical method to visualize inter-group metabolic differences, where each point represented an individual sample, and the distance between them reflected the scale of their metabolic differences. As the PCA score plot shown in **Figure 7**, the LPM group was clearly separated from the other three groups, indicating that marked alteration of plasma metabolites appeared in rats by PM-treated with LPS. While with middle dose of Poria treatment, the metabolic patterns of rats were closest to the Ctr, indicating recovery of metabolic feature and pathological improvements.

**FIGURE 8:**
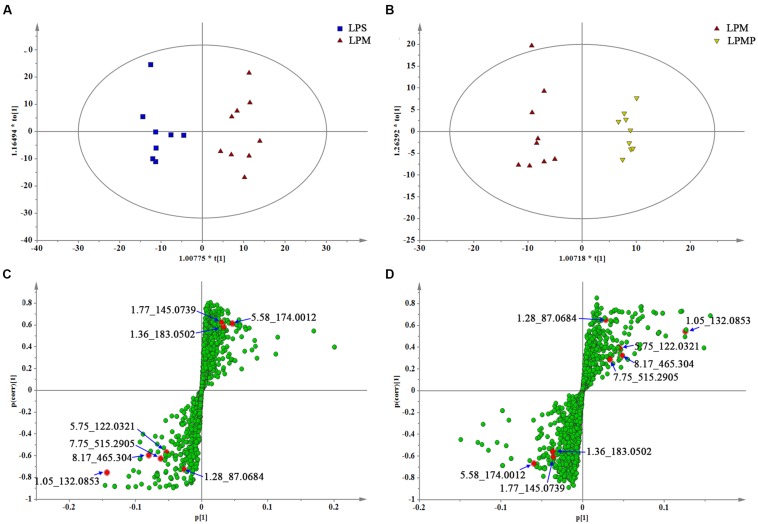
**The OPLS-DA score plots and S-plots generated from the OPLS-DA of the QTOF/MS data from LPS, LPM, and LPMP groups in the ESI- mode.** OPLS-DA score plots were the pair-wise comparisons between the LPS and LPM **(A)** as well as the LPM and LPMP **(B)**. S-plot of the OPLS-DA model were for the LPS and LPM **(C)** and the LPM and LPMP **(D)**, which axes are plotted in the S-plot from the predictive component are *p*1 vs. *p*(corr)1, representing the magnitude (modeled covariation) and reliability (modeled correlation), respectively. The points in red indicate the identified biomarkers.

Orthogonal partial least-square-discriminant analysis was applied to better understand the different metabolic patterns and gave rise to identify potential biomarkers that were significantly changed between LPS and herbs groups. Quality of resulting discriminant models were summarized in **Supplementary Table S2**. The key model parameters, *R*^2^ and *Q*^2^ in pair-wise groups were all larger than 0.5, suggesting robust fit and prediction of all the models. Score plots from the supervised OPLS-DA showed clear separation between the LPS and LPM groups, or the LPM and LPMP groups in both positive (**Supplementary Figure S1**) and negative ion modes (**Figures 8A,B**; **Supplementary Figures S1A,B**), suggesting a significant plasma biochemical perturbation in the LPM group. From the corresponding S-plots, variables further away from the origin were considered to contribute more significantly, thus they were more responsible for the separation between aforementioned groups. These variables may therefore be regarded as potential biomarkers (**Figures 8C,D**; Figures S1C,D). Variables determined by variable importance for projection (VIP) value >1 were pre-screened as potential biomarkers. To decrease the rate of false positives in the selection, variables with |*p*(corr)|≥ 0.5 were selected.

Co-metabolites that differed significantly (*p* < 0.05) in the LPM group compared with the LPS group as well as the LPMP group were selected as potential biomarkers. Criterion was restricted to feature with an average normalized intensity difference of 1.5-fold. Then, metabolites in the ESI+ and ESI- mode analyses were combined and subjected to further identification of their molecular formulas. All biomarkers were tentatively identified with the accurate mass charge ratio by the online METLIN database^1^. To determine the potential structures of the ions, targeted MS/MS analysis was applied to identify the metabolites. In the evaluation of the LPS and LPM groups to seek biomarker of PM-induced liver injury with non-injurious LPS in rats, fourteen metabolites were significantly (*p* < 0.05) altered, and they were summarized in **Table 1** with corresponding retention time, m/z, formula and trend. Intriguingly, the compatible proportion of PM and Poria 2:1 induced the most dramatic metabolic changes with thirteen metabolites of them. Through comparison, we found that ten of the fourteen biomarkers (creatine, arginine, sphingosine, sphinganine 1-phosphate, adrenoyl ethanolamide, ornithine, 4-aminobutyraldehyde, uridine, taurocholate, glycocholate) were significantly altered and even normalized compare to the Ctr. This result was consistent with the observed improvements in biochemical parameters and histological findings, suggesting the potential of these metabolites to be developed as biomarkers for improved liver damage.

**Table 1:** Differential metabolites for discrimination among lipopolysaccharide (LPS), LPS group treated with PM (LPM), and LPS group co-treated with PM + middle dose of Poria (LPMP) groups.

No.	Metabolite	Mass	tR/min	Formula	LPM/LPS			LPMP/LPM		
						
					VIP	Fold	*p*	VIP	Fold	*p*
**ESI+ mode**										
1	Creatine	131.0692	0.82	C4H9N3O2	1.67	0.63	0.0084	1.53	1.70	0.0254
2	Arginine	174.1124	0.93	C6H14N4O2	5.55	0.52	0.0052	5.76	1.92	0.0004
3	Sphingosine	299.2812	13.44	C18H37NO2	1.12	2.58	0.0086	1.11	0.45	0.0142
4	Sphinganine 1-phosphate	381.2663	19.72	C18H40NO5P	1.29	0.43	0.0194	1.21	2.08	0.0009
5	Disopyramide	339.2319	19.92	C21H29N3O	1.08	0.22	0.0140	0.93	3.63	0.0030
6	Adrenoyl ethanolamide	375.3123	21.08	C24H41NO2	1.61	0.47	0.0080	1.62	2.10	0.0009
**ESI- mode**										
7	Ornithine	132.0853	1.05	C5H12N2O2	5.83	0.48	0.0005	5.56	1.77	0.0106
8	4-Amino-butyraldehyde	87.0684	1.28	C4H9NO	1.01	0.37	0.0022	1.14	2.52	0.0049
9	4-Pyridoxic acid	183.0502	1.36	C8H9NO4	2.22	0.58	0.0168	1.92	1.55	0.0941
10	4-Acetamid-obutanoate	145.0739	1.77	C6H11NO3	2.44	0.52	0.0114	1.27	1.42	0.2873
11	Phenyl sulfate	174.0012	5.58	C6H6O4S	3.25	0.17	0.0400	1.72	3.36	0.7962
12	Uridine	122.0321	5.75	C9H12N2O6	1.47	2.06	0.0315	2.24	0.37	0.0141
13	Taurocholate	515.2905	7.75	C26H45NO7S	1.04	2.66	0.0460	1.32	0.32	0.0317
14	Glycocholate	465.3040	8.17	C26H43NO6	1.17	1.95	0.0491	1.33	0.52	0.0361


### Biological Pathway Analysis of Identified Biomarkers

**FIGURE 9:**
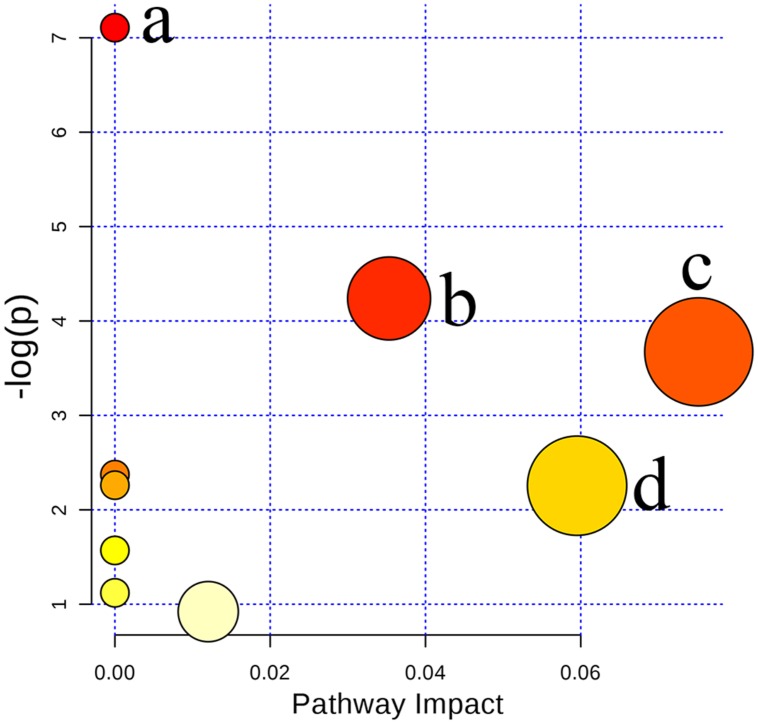
**Summary of pathway analysis with MetaboAnalyst 3.0: **(a)** Arginine and ornithine metabolism; **(b)** Arginine and proline metabolism; **(c)** Sphingolipid metabolism; **(d)** Primary bile acid biosynthesis**.

In the present study, ten metabolites were expressed at significantly different levels. Arginine, sphinganine 1-phosphate, adrenoyl ethanolamide, ornithine, creatine, and 4-aminobutyraldehyde were up-regulated while sphingosine, uridine, taurocholate, and glycocholate were down-regulated when Poria attenuated liver injury induced by PM/LPS in rats. To further reveal the relationship among these distinct biomarkers, we targeted nine potential metabolic pathways (**Supplementary Table S3**). Top three of importance included arginine and proline metabolism, primary bile acid biosynthesis and sphingolipid metabolism as shown in **Figure 9**.

.

## Discussion

Previous study has been proposed that inflammatory stress may render an individual with higher susceptible to idiosyncratic adverse drug reactions. Indeed, some idiosyncratic drug-induced liver injury (IDILI) have been developed in LPS/drug models that support this inflammatory stress hypothesis (Kaplowitz, 2005; Waring et al., 2006; Roth and Ganey, 2011). In the present study, our data indicated single Chinese herb (PM, Poria, Licorice, or Panax notoginseng) or LPS alone did not effectively increase the clinically used biomarkers of liver damage or cause any lesions in the liver as confirmed by biochemical and histological examinations. However, in PM/LPS rats, plasma markers of hepatocytes injury including ALT, AST, and TBA were increased significantly while none of Poria, Licorice or Panax notoginseng treated with LPS had the similar effect. These data vigorously proves that PM induces the idiosyncratic hepatotoxicity based on LPS model which is highly consistent with our team’s previous proposal of immunological stress-mediated idiosyncratic liver injury induced by PM (Wang et al., 2016).

Duality is a nature of most drugs with both wanted and unwanted effects at the same time. TCM is no exception. However, with the goal of counteracting toxicities and/or enhancing the therapeutic effects, drug compatibility has shown its unique significance to handle this problem in long-term clinical practices since ancient times. Interestingly, our compatibility test showed when co-treated with Poria, PM reversed its hepatotoxicity. In the meantime, Poria showed the best compatibility effect than any other candidates, especially with the optimal proportion of PM and Poria 2:1. This is the first study to ever report the reasonable compatibility of Poria could play a role of attenuating idiosyncratic toxicity induced by PM/LPS.

Recent evidences from experimental models indicate that mild inflammatory microenvironment may markedly increase the susceptibility of individuals to drugs with hepatotoxic effects and a toxic reaction would not occur in normal body (i.e., an “idiosyncratic” response) (Waring et al., 2006; Deng et al., 2009). In LPM group, levels of inflammatory cytokines increased markedly than any other groups while they were significantly ameliorated fully proved by plasma ELISA and liver RT-qPCR analyses. Besides, several studies have demonstrated anti-inflammatory activities of Poria. Specifically, Jeong et al. (2014) showed ethanol extract of Poria reduced the production of inflammatory mediators by suppressing the NF-kappaB signaling pathway in LPS-stimulated RAW 264.7 macrophages (Fuchs et al., 2006). As Poria played hepatoprotective effects against PM-induced liver injury in the inflammatory stress, it was reasonable to suggest that Poria about half dose of PM might diminish the liver susceptibility by improving the inflammatory microenvironment.

**FIGURE 10:**
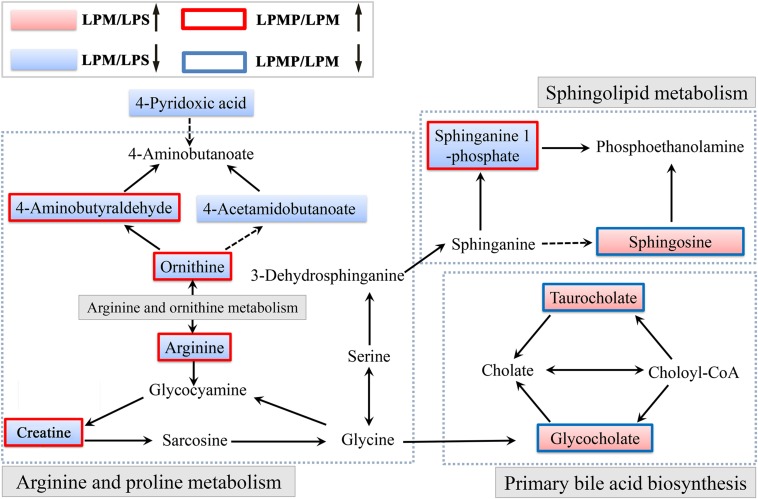
**Schematic diagram of the disturbed metabolic pathway related to PM and Poria treatment based on the LPS model.** (↑) in red represents metabolite increase and (↓) in blue is metabolites reduce. The related metabolic pathway is marked in gray bold.

Herbal medicines have recently been recognized as the major cause of drug-induced liver injury (DILI) (Wang et al., 2015). Herbs are often characterized as multi-component and multi-target. Thus, DILI diagnosis is difficult due to the lack of definitive operation standards and methods, let alone IDILI with the concealed feature. Metabolomics, focused on the comprehensive analysis of endogenous metabolites within the integrated biological systems, is a powerful technique to develop predictive biomarkers related to prognosis or diagnosis of a disease or drug toxicity/efficacy (Guma et al., 2016). Here, our LC-MS-based metabolomics investigation of the plasma revealed that metabolic perturbation occurred in LPM group and Poria co-administration attenuated this perturbation. Ten metabolites were expressed differentially among LPS, PM/LPS, and detoxification-treated groups. Affected metabolites were tightly correlated with arginine and proline metabolism, primary bile acid biosynthesis and sphingolipid metabolism. Summary of those proposed pathways was displayed in **Figure 10**. System analysis of metabolic networks that are a central paradigm in biology will help us to identify potential biomarkers for developing new diagnostic and therapeutic strategies and provide insights into molecular mechanisms of how Poria exerts effective treatment to PM/LPS-induced hepatotoxicity.

The liver is responsible for bile formation and biliary secretion (Schadt et al., 2016). Thus, any changes of bile acid homeostasis might be linked to various types of liver injury. In this study, plasma biochemical parameters reflected loss of hepatic functions with the significant elevation in the level of ALT, AST, and TBA, indicating that the hepatic injury caused by PM/LPS could influence bile acid metabolism. This was not surprising as liver disease has been reported to disturb the synthesis, metabolism, clearance and intestinal absorption of hepatic bile acids (Bobeldijk et al., 2008; Alnouti, 2009). Comparing LPS group with LPM in metabolites, the levels of taurocholate and glycocholate derived from TBA rose significantly, while which reduced greatly in LPP group, demonstrating the efficacy of Poria in ameliorating the changes of TBA metabolites. In fact, Luo et al. have evaluated cholic acid, taurocholate, and glycocholate as potential biomarkers of liver injury in rodent models by a targeted LC-MS/MS approach to differentiate various types of liver injury (Luo et al., 2014). Therefore, alterations in TBA profiles can be the consequence of hepatotoxicity. In addition, Allen et al. showed bile acids could induce up-regulation of inflammatory genes expression in hepatocytes after bile duct ligation, which may promote hepatic inflammation during obstructive cholestasis and contribute to DILI (Allen et al., 2011).

Arginine, a versatile amino acid in animals, serves as a precursor for ornithine, urea, nitric oxide, and creatine. It has been suggested as a biomarker candidate for liver injury which has been found decreased in hepatic injuries by hepatotoxicants such as thioacetamide and carbon tetrachloride (Saitoh et al., 2014). Rats in LPM group exhibited significant reduction of plasma creatine, arginine, ornithine, and 4-aminobutyraldehyde, suggesting a disturbance of arginine and ornithine metabolism. Indeed, Saitoh et al. (2014) confirmed that arginine-to-ornithine pathway was altered in association with acute hepatic injury, and plasma arginine and ornithine were possibly specific biomarkers for liver injury. Interestingly, creatine synthesis is initiated by arginine glycine amidinotransferase (AGA) that transforms arginine to ornithine. Since AGA has been reported to be located in the liver, the decreased blood creatine levels might contribute to the alterations in blood arginine and ornithine following liver injury (Wu and Morris, 1998). Nicholson et al. also demonstrated creatine and creatinine could reflect the injury of both liver and kidney (Nicholson et al., 2002). Additionally, inflammatory stimuli (e.g., LPS) greatly stimulated the expression of arginase I, arginase II, and ornithine decarboxylase in many cell types (Morris, 2007). Therefore, these increased substances upregulated arginine metabolism for the synthesis of urea, ornithine, proline, and polyamines in response to inflammation. In contrast, concentrations of arginine, ornithine and 4-aminobutyraldehyde in LPM plasma were reduced markedly suggesting the disorder of arginine and ornithine metabolism.

Sphingolipids are critical regulators of hepatic homeostasis and contribute to the progress of several liver diseases (Mari and Fernandez-Checa, 2007). They are structural components of biological membranes bilayer presenting as a diverse class of lipids including free sphingoid bases and their phosphates such as sphingosine and sphinganine-1-phosphate (Grammatikos et al., 2014). Recently, sphinganine-1-phosphate has been reported as a mediator of liver regeneration after injury (Nojima et al., 2015). In this study, the decreased concentration of sphinganine-1-phosphate and the rising of sphingosine in the LPM group signified that the LPM-injured rats might lose the homeostasis of normal metabolite. On the contrary, the concentrations of sphinganine-1-phosphate and sphingosine were recovered in LPP group, and the liver of rats could be concomitantly protected. Moreover, TNF-α and IL-1β are important mediators of liver inflammation and injury (Chen et al., 1995). Studies indicate that the acyl chain length and composition in sphingolipids play a crucial role in regulating internalization of the TNF-α receptor-1 and subsequent apoptosis signaling pathways in hepatocytes (Albi et al., 2013). Ceramide generation could be stimulated by IL-1β in hepatocytes (Chen et al., 1995), whose accumulation has been implicated as an important contributing factor of liver injury and apoptosis. In sum, the initiation and progression of liver injury is multifactorial, and understanding of sphingolipid metabolic pathway may open up a novel therapeutic avenue to liver diseases.

## Conclusion

Consistent with previous studies, we showed PM could induce idiosyncratic hepatotoxicity in rats with modest inflammatory episode triggered by non-injurious dose of LPS. Secondly, the proper proportion of PM and Poria 2:1 effectively attenuated idiosyncratic toxicity induced by PM/LPS. Finally, ten potential biomarkers involved in three metabolic pathways including arginine and proline metabolism, primary bile acid biosynthesis and sphingolipid metabolism were identified by metabolomics. Among these, inflammatory stress played an important role in the initiation and progression of liver injury. The current study provides the very first systematic experimental basis of the hepatoprotective effects of Poria against PM/LPS-induced liver injury. Although further studies are needed to validate diagnostic and/or prognostic values of the identified potential biomarkers, the present strategy provided insights into studying the multifactorial molecular mechanisms of how Poria exerts effective treatment to PM/LPS-induced liver injury.

## Author Contributions

K-JZ, W-WG, J-BW, and X-HX conceived and designed the experiments. DG, J-YP, and C-EZ performed the investigation, analyzed the data and wrote the paper. C-YL, CT, and H-ZZ contributed to reagents/materials/experiments. MN and YX amended the paper.

## Conflict of Interest Statement

The authors declare that the research was conducted in the absence of any commercial or financial relationships that could be construed as a potential conflict of interest.

## Footnotes

1 https://metlin.scripps.edu/

2 http://www.hmdb.ca/

3 http://www.genome.jp/kegg/

## Supplementary Material

The Supplementary Material for this article can be foundonline at: http://journal.frontiersin.org/article/10.3389/fphar.2016.00386
