# Supplementary material for: Poria Attenuates Idiosyncratic Liver Injury Induced by Polygoni Multiflori Radix Praeparata
Source: Front Pharmacol. 2016 Oct 18;7:386. doi: 10.3389/fphar.2016.00386 (PMC5067826; doi:10.3389/fphar.2016.00386)
Supplement: Supplementary file 1 [file Presentation_1.PDF]

Supplementary materials for：Metabolomics Revealed the Hepatoprotective Effects of Poria against Polygoni Multiflori Radix Praeparata-induced Idiosyncratic Liver Injury.

Dan Gao,[1,2‡] Jing-yao Pang,[3,4‡] Cong-en Zhang,[2‡] Chun-yu Li,[1,2] Can Tu,[2] Hai-zhu Zhang,[2] Ming Niu,[2] Yin Xiong,[5] Xiao-he Xiao,[6] Kui-jun Zhao,[4*] Wei-wei Gao,[1*] Jia-bo Wang[2*]

Detailed operations of TUNEL assay

Sections were dewaxed in xylene, rehydrated as follows: twice in xylene bath for 20 min each, twice in 100% ethanol for 10 min each, and then for 5 min each in graded alcohols (100%, 95%, 90%, 80%, 70%) and three times in water. The slides were then digested by incubation with 5 μg/ml proteinase K for 15 min, and washed three times in phosphate buffered saline (PBS) for 5 min each. Peroxidase and DNAses were inactivated by covering the sections with 3% H_2O_2 in methanol for 20 min, rinsed three times with PBS in the dark. Thereafter, the sections were incubated for 60 minutes in a humidified chamber at 37±℃ with 2:29 diluted terminal deoxynucleotidyl transferase (TDT) in the reaction buffer that contained digoxigenin-11-dUTP. Stop-wash buffer was used for 10 minutes to stop the reaction. After washing with PBS, the peroxidase-DAB reactions visualize the apoptotic nuclei and the slides were counterstained with hematoxylin.

RNA isolation and real-time RT-PCR

Primer sequences were designed using Primer Express (AB) shown in Table S1.

Table S1 Primer sequences for RT-qPCR assays

Gene	Primer sequence (5' to 3')	Length(bp)
IL-6	Forward: 5’-ACCACCCACAACAGACCAGT-3’	20
	Reverse: 5’ -ACAGTGCATCATTCGCTGTTC-3’	
IL-12-p40	Forward: 5’ -ATGTGGGAGCTGGAGAAAGA-3’	20
	Reverse: 5’ -CCACGTCTCTGGTCTGAGGT-3’	
IFN-γ	Forward: 5’ -AAAGACAACCAGGCCATCAG-3’	20

	Reverse: 5’-CTGGATCTGTGGGTTGTTCA-3’	
TNF-α	Forward: 5’-GGTCCCAACAAGGAGGAGA-3’	19
	Reverse: 5’-GGGCTTGTCACTCGAGTTTT-3’	
IL-27	Forward: 5’-CCCCAATGTTTCCCTGACCTTCC-3’	23
	Reverse: 5’-ATTGCCCACAGCTGCTCCCTCTC-3’	
IL-1β	Forward: 5’-CCTGTTCTTTGAGGCTGACA-3’	20
	Reverse: 5’-GCTGTGAGATTTGAAGCTGGA-3’	
GAPDH	Forward: 5’-ACCACAGTCCATGCCATCAC-3’	21
	Reverse: 5’- TCCACCACCCTGTTGCTGTA-3’	

UPLC-MS analysis

Metabolite separation was conducted on an Agilent 6550 iFunnel Q-TOF LC/MS (Agilent Technologies, USA). Chromatography was performed on an Agilent ZORBAX Eclipse Plus C18 600 column (2.1X100 mm, 1.8 μm). A “purge-wash-purge” cycle was employed on the autosampler, with 90% aqueous formic acid used for the wash solvent and 0.1% aqueous formic acid used as the purge solvent; this ensured that the carry-over between injections was minimized. The column was maintained at 30 ℃, and subsequently, the mobile phase was composed of 0.1% formic acid in acetonitrile (solvent A) and 0.1% formic acid in water (solvent B) with a linear gradient elution: 0−1.0 min, 1-5% B; 1.0−9.0 min, 5 −40% B; 9.0−19.0 min, 40−90% B; 19.0−25.0 min, 90−100% B; then 100% B kept for 2.0 min, returned to 1% B for 5 min recycle time. The flow rate was 0.30 mL min^{-1}, and 4 μL aliquot of each sample was injected onto the column. The eluent was introduced to the mass spectrometry directly without split. A high resolution electrospray mass spectrometer (MicrOTOF-QII, Bruker Daltonics Corporation, USA) was operated in both positive and negative ion mode using electrospray ionization (ESI). Data were collected in the full scan mode from m/z 50 to m /z 1000. The optimal conditions of analysis were as follows: the capillary voltage and the cone voltage were set at 4000 V and 45 V respectively, the desolvation gas flow was 11 L/min at a temperature of 225°C, the MS interface capillary was maintained at 350 ℃ with a sheath gas flow of 12 L/min. After every 10 sample injections, a pooled sample followed by a blank was injected in order to ensure consistent performance of the system.

Table S2 Parameters of PCA and OPLS-DA models

Model	Model	Groups	Component	R^2X	R^2Y	Q^2Y

No.	type	included			(cum)	(cum)
Data of ESI+ mode						
M1	PCA	N, L, LS & LSW	6	0.568	—	0.107
M2	OPLS-DA	L & LS	1+1+0	—	0.887	0.515
M3	OPLS-DA	LS & LSW	1+1+0	—	0.937	0.690
Data of ESI- mode						
M4	PCA	N, L, LS & LSW	3	0.567	—	0.183
M5	OPLS-DA	L & LS	1+0	—	0.926	0.789
M6	OPLS-DA	LS & LSW	1+1	—	0.971	0.802

Table S3 Result from pathway analysis with MetaboAnalyst 3.0

Pathway name	Total	Hits	Raw *p*	-log(*p*)	FDR	Impact
Arginine and ornithine metabolism	4	2	0.0008	7.1074	0.07	0.00
Arginine and proline metabolism	44	3	0.0144	4.2388	0.58	0.04
Sphingolipid metabolism	21	2	0.0254	3.6734	0.69	0.08
Taurine and hypotaurine metabolism	8	1	0.0932	2.3729	1.00	0.00
Vitamin B6 metabolism	9	1	0.1043	2.2608	1.00	0.00
Primary bile acid biosynthesis	46	2	0.1048	2.2553	1.00	0.06
beta-Alanine metabolism	19	1	0.2081	1.5698	1.00	0.00
Glycine, serine and threonine metabolism	32	1	0.3262	1.1203	1.00	0.00
Pyrimidine metabolism	41	1	0.3980	0.9213	1.00	0.01

Total is the total number of compounds in the pathway; the hits is the actually matched number from the user uploaded data; the raw *p* is the original *p* value calculated from the enrichment analysis; the impact is the pathway impact value calculated from pathway topology analysis.

For the less obvious TUNEL stain which might result from the bad background color weakened the image stand out from this page background, we enlarged the image a bit. Showed as following **Figure r1**, a large number of cells stained positively in the TUNEL reaction after PM/LPS administered and middle- (0.54g/kg) dose of Poria inhibited apoptosis better rarely detected hepatocyte apoptosis than others (**Figure r1C, H**). Besides, high-dose of Poria-treated with LPS led to some positive TUNEL reaction (**Figure r1D**), which perhaps because excessive doses entered in the liver metabolism would increase liver burden.

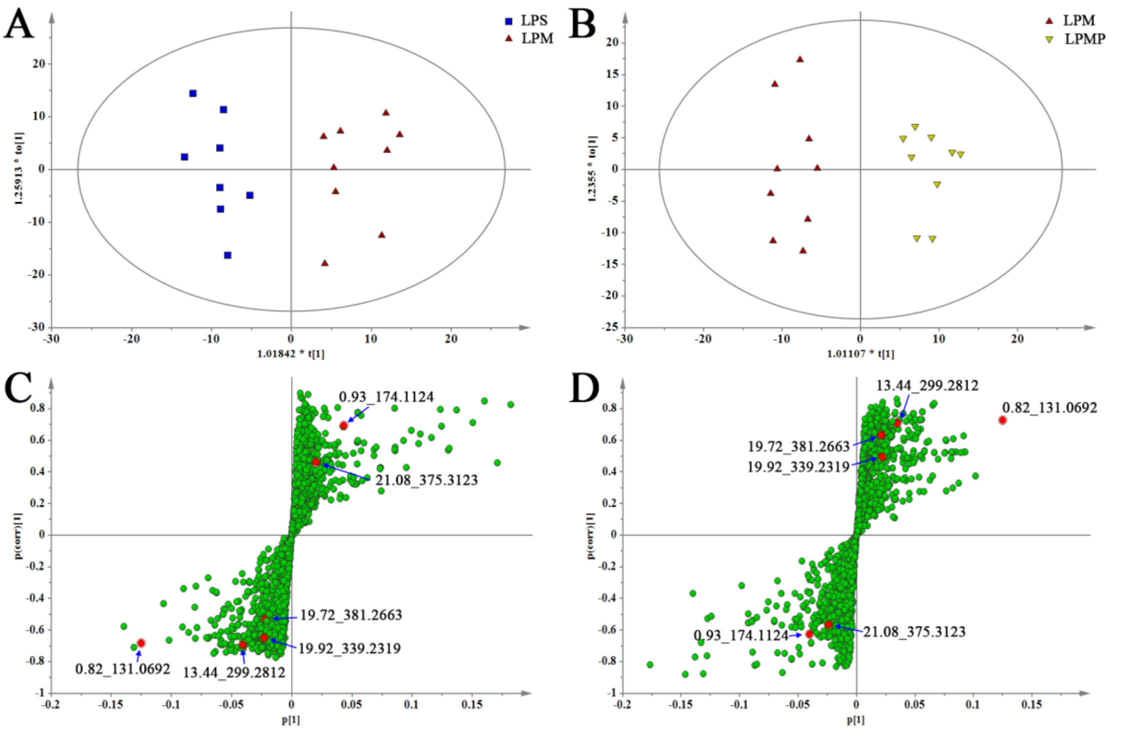

Figure 1S The OPLS-DA score plots and S-plots generated from the OPLS-DA of the QTOF/MS data from LPS, LPM and LPMP groups in the ESI+ mode. OPLS-DA score plots were the pair-wise comparisons between the LPS and LPM (A) as well as the LPM and LPMP (B). S-plot of the OPLS-DA model were for the LPS and LPM (C) and the LPM and LPMP (D), which axes are plotted in the S-plot from the predictive component are p1 vs. p(corr)1, representing the magnitude (modeled covariation) and reliability (modeled correlation) respectively. The points in red indicate the identified biomarkers.

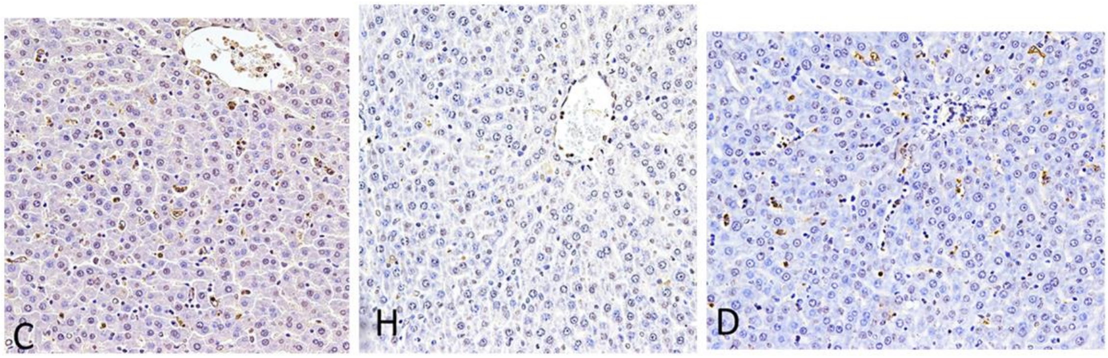

Figure r1 Hepatocyte apoptosis in rat by TUNEL assay (enlarged a portion of Figure 6). C: PM treated with LPS group (LPM), D: high dose of Poria treated with LPS group (LHP), H: PM + middle dose of Poria co-treated with LPS group (LPMP).
